# Graph-aware spatio-temporal attention for forecasting HIV/AIDS case counts in public health surveillance

**DOI:** 10.3389/fpubh.2026.1771586

**Published:** 2026-03-25

**Authors:** Ziyue Wang, Hossein Khaleghy, Yisong Yao, Jia Chen

**Affiliations:** 1 Xiangya School of Nursing, Central South University, Changsha, China; 2 School of Nursing and Midwifery, University of Galway, Galway, Ireland; 3 School of Computer Science, University of Galway, Galway, Ireland; 4 Key Laboratory of Digital-Intelligent Disease Surveillance and Health Governance, North Sichuan Medical College, Nanchong, China

**Keywords:** 3DCNN, AIDS/HIV spatio-temporal forecasting, attention mechanism, inter-provincial graph structure, public health surveillance

## Abstract

**Introduction:**

Provincial-level monthly HIV/AIDS case-count forecasting is important for public health surveillance, early warning, and resource allocation. Existing methods often struggle to capture both cross-regional dependency patterns and complex temporal dynamics from routine notification data.

**Methods:**

Using province-by-month case reports from January 2010 to December 2020, a graph-aware spatio-temporal forecasting framework was developed. The model integrates multi-scale 3DCNN encoding, Graph-Aware Spatio-Temporal Attention for modeling inter-provincial relations on a province graph, and a Seasonal Structure-Aware Temporal Module to represent trend and multi-period seasonality.

**Results:**

The proposed method achieved better predictive accuracy and spatial consistency than representative baseline models, with MSE of 12.5336, MAE of 1.4546, SSIM of 0.889, and PSNR of 32.74. Ablation experiments further showed that graph-aware attention and seasonal structure modeling each contributed to performance improvement.

**Discussion:**

The results indicate that jointly modeling inter-provincial dependency and seasonal temporal structure can improve provincial HIV/AIDS forecasting. This framework provides a useful pathway for more reliable spatial-temporal surveillance and decision support in public health practice.

## 1 Introduction

Since the widespread adoption of antiretroviral therapy has substantially reduced HIV-related mortality and extended life expectancy, HIV/AIDS has gradually transitioned from an acute infectious disease to a chronic condition requiring long-term management. Nevertheless, it continues to impose a substantial public health burden at both global and regional levels, particularly in resource-limited settings and among key populations, where pronounced disparities in incidence and mortality persist (1). In recent years, the continuous improvement of surveillance systems in China and other low- and middle-income countries has enabled epidemiological analyses based on routinely collected monitoring data.

A growing body of evidence has demonstrated marked spatio-temporal heterogeneity and clustering in HIV epidemics across regions and sub-populations, including studies on regional variation and recent infection patterns in Sichuan Province (2), as well as spatio-temporal analyses of program indicators in northwestern Ethiopia (3). At the same time, increasingly fine-grained investigations targeting specific high-priority regions and populations have emerged, such as Bayesian spatio-temporal analyses of HIV/AIDS distribution and associated factors among young populations in Guangxi, China (4), and spatio-temporal estimation of risk group proportions among adolescent girls and young women in selected sub-Saharan African countries (5). Collectively, these findings indicate that reliance on aggregate statistics alone is insufficient to support precision prevention and resource allocation, underscoring the urgent need for high-resolution spatio-temporal prediction tools.

From a methodological perspective, traditional statistical approaches and Bayesian hierarchical models have been widely applied to characterize spatial clustering, temporal trends, and structural determinants of HIV epidemics. Representative examples include Bayesian latent structural equation models for syndemic mapping of HIV and other sexually transmitted infections in KwaZulu-Natal, South Africa (6), as well as R-INLA-based spatio-temporal analyses of HIV/AIDS prevalence patterns and risk factors in Zhejiang Province, China, from 2005 to 2022 (7). At broader temporal horizons and larger spatial scales, researchers have integrated surveillance data with projection models to examine spatial variation, temporal dynamics, and medium- to long-term trends in HIV/AIDS incidence across eastern China (8). Similar county-level spatio-temporal analyses in the United States have been conducted to assess the alignment between new HIV diagnoses and prevention services, such as pre-exposure prophylaxis coverage (9). Comparative studies further suggest that explicitly modeling spatial dependence can substantially improve predictive performance relative to non-spatial alternatives in county-level HIV forecasting tasks (10). Meanwhile, international HIV estimation and projection frameworks, such as the continually updated Spectrum case surveillance and mortality tools, have advanced population-level estimation methodologies, yet they primarily focus on national or large regional aggregates and remain limited in capturing fine-grained interregional interactions and complex network structures (11). However, these model families typically rely on pre-specified spatial smoothness assumptions or coarse interaction structures, which can limit their ability to represent heterogeneous interregional coupling and evolving transmission-related dependencies in routine surveillance settings.

With the increasing volume and dimensionality of surveillance data, time-series models and machine learning techniques have been progressively introduced into HIV prediction tasks to enhance the characterization of trends in case counts and treatment coverage. For example, studies in Pakistan have evaluated multiple statistical time-series and machine learning models for forecasting HIV and antiretroviral therapy case numbers, systematically comparing their predictive performance (12). In selected regions of Ethiopia, deep learning models have been explored to forecast HIV infection trends in the presence of nonlinear dynamics and multifactorial drivers (13). In parallel, emerging computational paradigms, such as quantum algorithms, have been investigated for their potential in healthcare prediction and complex system modeling, offering new perspectives for high-dimensional epidemiological analysis (14). However, from a public health decision-support standpoint, existing approaches are often confined either to purely temporal modeling or to limited spatial dependency structures, and remain insufficient for simultaneously capturing interregional relationships, potential transmission pathways, and multi-scale seasonal patterns within a unified graph-based framework. In particular, routine province-level case notification series often exhibit long-horizon seasonality, abrupt regime shifts, and spatially uneven reporting intensity, posing challenges that are not fully addressed by purely temporal predictors or spatially simplified models.

In the broader field of infectious disease modeling, recent advances in spatio-temporal graph neural networks and attention-based architectures have demonstrated notable advantages across a range of epidemic forecasting scenarios. Representative work includes the Spatio-Temporal Attention Network (STAN), which leverages explicit spatio-temporal attention mechanisms to improve the representation of epidemic trajectories based on real-world evidence (15), as well as adaptive temporal graph convolutional models designed to capture age-structured contact patterns across multiple demographic groups (16). In the context of COVID-19 and other respiratory infectious diseases, spatio-temporal graph neural networks have been employed to model regional SARS-CoV-2 transmission dynamics in the Netherlands, showing significant improvements over traditional baselines in regional-level case prediction tasks (17). Although recent studies have begun to explore the integration of spatio-temporal graph learning with epidemiological factors for short-term HIV forecasting (18), existing approaches generally do not explicitly address the long-term seasonal structure of HIV/AIDS case counts, the multi-layered interregional connectivity, or the inherent imbalance and heterogeneity of surveillance data. To address these limitations, this study proposes a Graph-Aware Spatio-Temporal Attention framework incorporating a Seasonal Structure-Aware Temporal Module, aiming to enable high-resolution spatio-temporal forecasting of HIV/AIDS case counts in public health surveillance settings, and to provide interpretable and scalable methodological support for hotspot identification, resource allocation optimization, and intervention strategy design. Compared with existing HIV forecasting research, the proposed methodology makes three targeted contributions: (1) it introduces a graph-constrained spatio-temporal attention mechanism that explicitly leverages an inter-provincial adjacency structure to learn transmission-related cross-region dependencies beyond purely temporal modeling; (2) it integrates a seasonal structure-aware temporal module that performs trend–seasonality decomposition and adaptive fusion to stabilize long-horizon prediction under multi-scale periodic fluctuations; (3) it outputs geographically consistent provincial risk distributions and evaluates them using both numerical error and spatial fidelity metrics, providing an interpretable and decision-oriented forecasting pipeline for public health surveillance.

## 2 Methods

### 2.1 Data collection and processing

#### 2.1.1 Data collection

**Figure 1:**
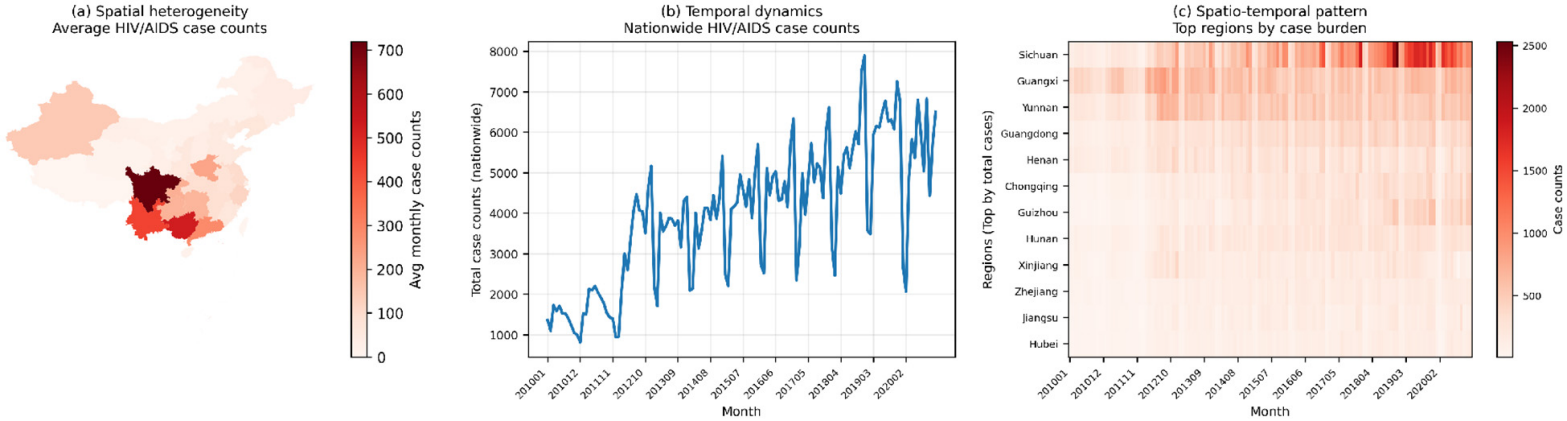
Overview of the spatio-temporal HIV/AIDS surveillance dataset used in this study: **(a)** summarizes the average provincial case counts over 2010–2020, **(b)** shows the nationwide monthly totals, and **(c)** displays a month–region heatmap for provinces with the highest cumulative burden. Together, these three views highlight pronounced spatial heterogeneity, strong temporal variability, and concentrated high-burden regions, which jointly motivate the development of graph-aware spatio-temporal forecasting models.

The data used in this study were obtained from the publicly available surveillance dataset released by the China Public Health Science Data Center, covering monthly HIV/AIDS case counts reported by provincial-level administrative regions from January 2010 to December 2020. Specifically, de-identified case summary tables were downloaded from the original platform by month and administrative unit. For each province and each calendar month, we extracted the number of newly reported cases and converted all records into a standardized structured format of *Time (YYYYMM) – Region – Case Count*. Subsequently, we conducted systematic data auditing and cleaning to address inconsistencies in region codes across years, missing entries, and extreme outliers, ensuring a continuous temporal axis and consistent spatial unit definitions. Importantly, the dataset contains only de-identified, aggregated monthly case summaries at the provincial level and does not include any individual-level records or personally identifiable information. Accordingly, the analyses were conducted solely on publicly available, aggregated surveillance notifications and therefore fall outside human-subject research requirements under the applicable governance framework. This procedure yielded high-quality and comparable data to support subsequent spatio-temporal modeling and forecasting analyses. A simplified example of the dataset is illustrated in Figure 1.

#### 2.1.2 Data processing

During the data preprocessing stage, the original surveillance tables from different years were first consolidated into a single master file, all_data.csv. Key fields, including region, time, and case count, were then standardized and cleaned by removing extraneous whitespace, converting case counts to numeric format, and discarding missing or invalid records. For missing values, we first checked whether region or time was absent; such records were removed because they cannot be reliably aligned to the spatial and temporal grid. For case counts, missing entries were imputed using linear interpolation within each province along the monthly timeline when gaps were short; if interpolation was not feasible at the sequence boundaries, the nearest valid observation within the same province was used. For outliers, we applied a robust, province-wise detection strategy: monthly case counts exceeding the median plus three times the interquartile range were flagged, and then winsorized to the corresponding threshold to reduce the influence of extreme spikes while preserving the overall trend. Subsequently, the aggregated “national” level was excluded, and only provincial-level administrative units were retained as spatial analysis units. To ensure consistent naming, suffixes such as “Province,” “City,” “Autonomous Region,” and “Special Administrative Region” were systematically removed using string-based rules to construct normalized provincial name fields.

**Figure 2:**
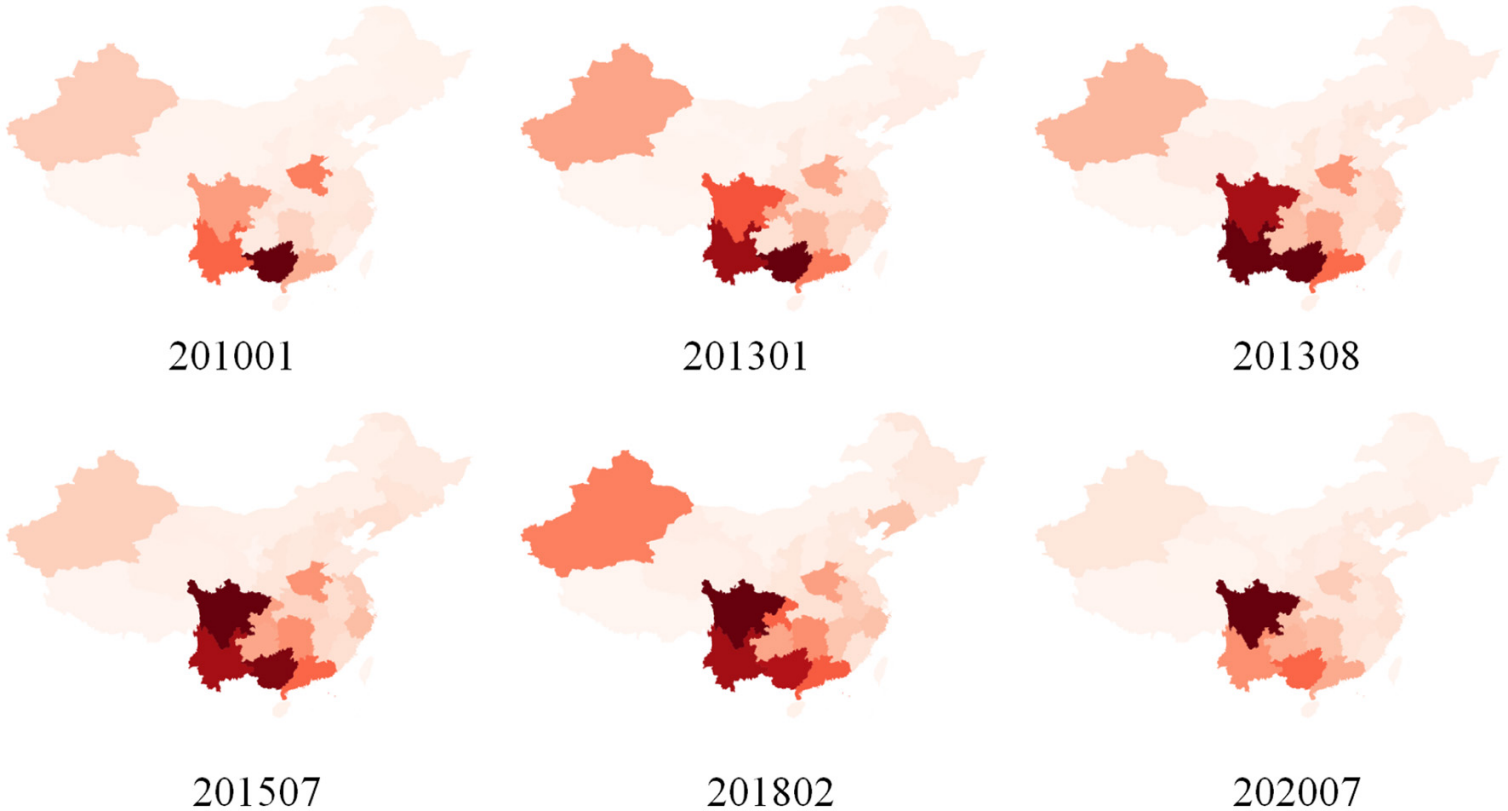
Example of a heatmap of the dataset used in the experiment.

To align the epidemiological records with spatial boundary data, provincial names were further matched and corrected against a China provincial-level GeoJSON vector file provided by Aliyun. The cleaned data were then aggregated along the Time (YYYYMM)—Province dimension to obtain a matrix of newly reported cases for each province and each month. Heatmap generation was performed deterministically from this matrix using the same provincial polygon set and a fixed rasterization resolution across all months, with each province filled by its monthly case-count value and all non-province background pixels set to zero. Before rasterization, case counts were normalized using min–max scaling computed only on the training period, and the resulting scaling parameters were then reused unchanged for validation and test months to prevent any information leakage. To ensure comparable color intensity across months and provinces, we applied a single global color scale controlled by the training-derived minimum and maximum, and we did not rescale colors separately per month. All heatmaps were rendered without axes, legends, or annotations, and were saved as PNG files with consistent image size and value-to-color mapping, so that identical numeric inputs yield identical visual outputs. Based on this matrix, monthly provincial case counts were mapped onto the corresponding spatial polygons, generating a series of province-level incidence heatmap PNG images without axes or legends. These images were used both as spatial-view inputs for subsequent spatio-temporal modeling and as the basis for exploratory visualization analyses. An illustrative example of the processed data is shown in Figure 2.

### 2.2 Problem Definition

This study focuses on the spatio-temporal forecasting of reported HIV/AIDS case counts at the provincial level. Let *N* denote the number of provincial-level administrative regions, forming a node set *V* = {*v*_1_, …, *v*_*N*_}, and consider natural months as the temporal unit, spanning *T* time steps from January 2010 to December 2020. Let $x_{t}^{i}$ represent the reported case count at node *v*_*i*_ and time step *t*, and define the observation sequence as $X={\left\{X_{t}\right\}}_{t=1}^{T}$, where $X_{t}={\left[x_{t}^{1},\ldots ,x_{t}^{N}\right]}^{⊤}\in {\mathbb{R}}^{N}$. A static undirected graph *G* = (*V, E, A*) is constructed based on land adjacency relationships among provinces, where an edge (*i, j*)∈*E* exists if provinces *v*_*i*_ and *v*_*j*_ share a common boundary. The adjacency matrix *A*∈{0, 1}^*N*×*N*^ satisfies *a*_*ij*_ = 1 if nodes *v*_*i*_ and *v*_*j*_ are adjacent and *a*_*ij*_ = 0 otherwise, with self-loops included such that *a*_*ii*_ = 1. The graph structure is purely geographic and remains fixed over time, while the historical epidemic intensity is encoded only in the node-wise observation sequence 𝒳_*t*−*L*+1:*t*_ rather than in edge weights. For message passing convenience, we optionally apply symmetric normalization $Ã=D^{-\frac{1}{2}}AD^{-\frac{1}{2}}$, where *D* is the degree matrix of *A*. At any reference time *t*, given a historical observation window of length *L*, denoted by 𝒳_*t*−*L*+1:*t*_ = {*X*_*t*−*L*+1_, …, *X*_*t*_}, the objective is to learn a parameterized mapping *f*_θ_ under a fixed prediction horizon *H* that outputs the predicted provincial case count sequences for the subsequent *H* months, ${\hat{Y}}_{t+1:t+H}=f_{\theta }\left({𝒳}_{t-L+1:t},G\right)$, where ${\hat{Y}}_{t+1:t+H}=\left\{{\hat{X}}_{t+1},\ldots ,{\hat{X}}_{t+H}\right\}$ and ${\hat{X}}_{\tau }\in {\mathbb{R}}^{N}$ denotes the predicted case vector at time step τ. By minimizing the discrepancy between predicted and observed case counts over all sliding windows in the training period, using a loss function based on mean squared error and related evaluation metrics, the learned mapping *f*_θ_ enables fine-grained multi-step spatio-temporal forecasting of future HIV/AIDS case counts at provincial resolution given node-wise historical case burdens and a fixed border-adjacency graph.

### 2.3 Overall model architecture

**Figure 3:**
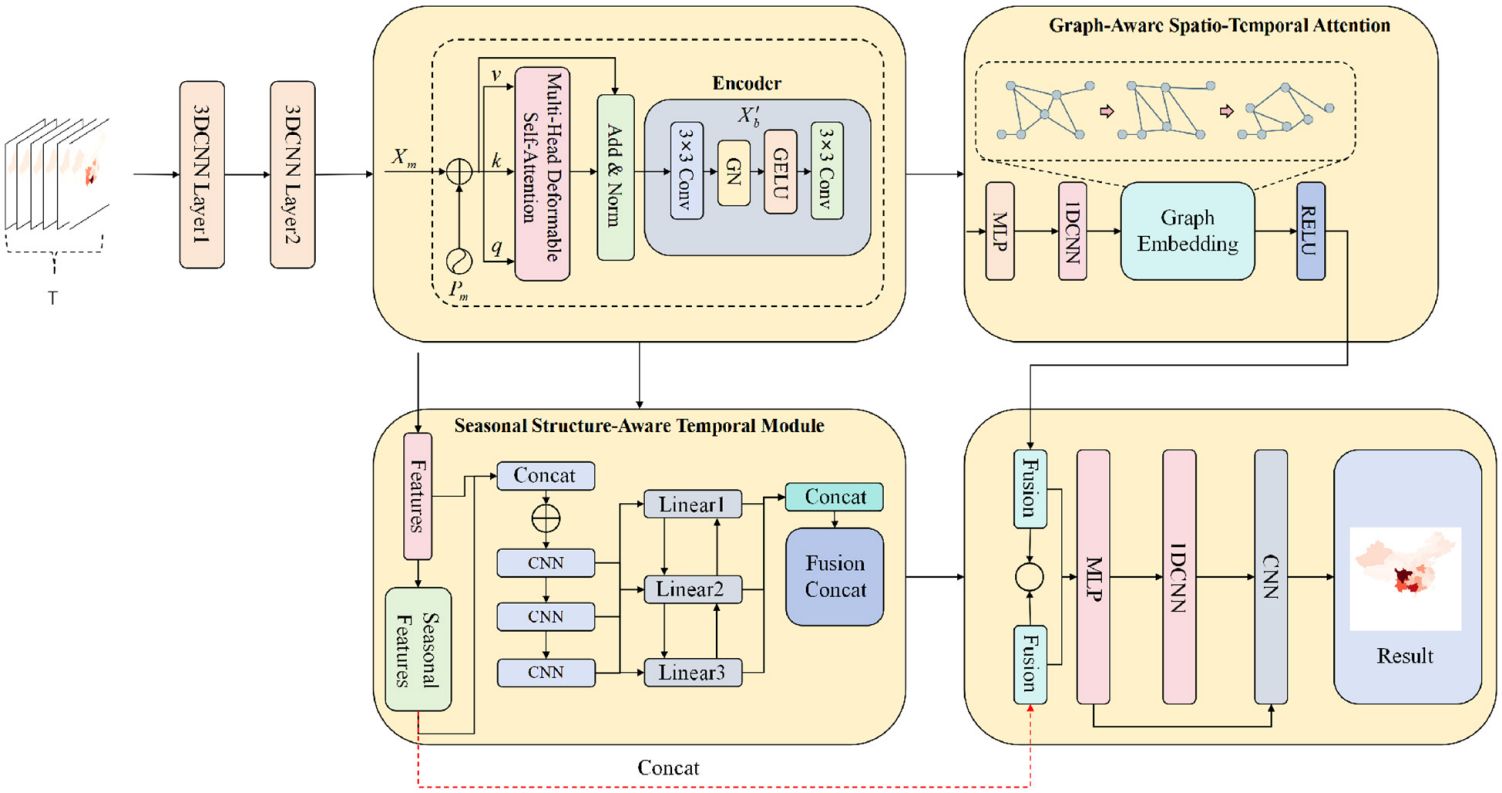
Overall architecture of the proposed HIV/AIDS spatio-temporal forecasting model, which first encodes a sequence of historical case-count heatmaps via stacked 3D CNN layers and a convolutional encoder. A graph-aware spatio-temporal attention module operating on a static undirected border-adjacency graph (without intensity-based edge weighting) and a seasonal structure-aware temporal module then jointly refine the representations, whose fused features are decoded by MLP, dilated CNN and 2D CNN blocks to generate future provincial-level case-count heatmaps.

The overall architecture of the proposed model is illustrated in Figure 3, aiming to learn stable spatio-temporal representations from a continuous sequence of provincial HIV/AIDS incidence heatmaps and to perform multi-step forward forecasting. First, a historical observation window of length *L*, {**I**_*t*−*L*+1_, …, **I**_*t*_}, is organized into a four-dimensional tensor ${\text{I}}_{t-L+1:t}\in {\mathbb{R}}^{L\times H\times W\times 1}$, where *H* and *W* denote the rasterized spatial resolution. A 3D convolutional encoder, composed of two stacked 3D CNN layers, jointly models the temporal and spatial dimensions and produces an intermediate feature tensor as shown in Formula 1:


$$\begin{array}{l} {\text{F}}_{t}=f_{3\text{DCNN}}\left({\text{I}}_{t-L+1:t}\right)\in {\mathbb{R}}^{L\times C\times H^{\prime }\times W^{\prime }}, \end{array}$$ (1)

where *C* is the number of channels and *H*′, *W*′ are the compressed spatial sizes. Subsequently, lightweight convolution and normalization operations are applied to compress **F**_*t*_ into a month-indexed feature sequence **Z**_*t*_ = {**z**_*t*−*L*+1_, …, **z**_*t*_}, where each time-step feature ${\text{z}}_{\tau }\in {\mathbb{R}}^{D}$ serves as the shared input to both the graph-aware spatio-temporal attention branch and the seasonal structure-aware temporal module, ensuring that the two branches operate collaboratively within a unified representation space.

In the graph-aware spatio-temporal attention branch, a static undirected graph *G* = (*V, E, A*) is constructed purely from administrative border adjacency relations, where *A* is a fixed binary adjacency matrix (with self-loops) and is not weighted or modified by epidemic intensity. A multi-layer graph embedding network encodes node-level structural information into an embedding matrix $\text{E}\in {\mathbb{R}}^{N\times d_{g}}$. The temporal features **Z**_*t*_ are then projected into query, key, and value matrices **Q**, **K**, **V**, and multi-head self-attention is performed under graph-structured constraints to obtain a spatio-temporal graph representation that enforces border-adjacency constraints while leveraging node-wise temporal context (i.e., historical case burden encoded in **Z**_*t*_) exclusively through node features rather than edge construction,


$$\begin{array}{l} {\text{H}}_{t}^{\text{G}}=\text{MHA}\left(\text{Q},\text{K},\text{V};A,\text{E}\right)\in {\mathbb{R}}^{L\times N\times d}, \end{array}$$ (2)

As shown in Formula 2, where the attention weights are non-zero only for node pairs permitted by the adjacency matrix *A*, thereby approximating inter-provincial interactions under fixed border-adjacency constraints. In parallel, within the seasonal structure-aware temporal module, the sequence **Z**_*t*_ is processed by a temporal network composed of multi-scale dilated convolutions and channel-wise linear transformations to separately extract annual periodicity, semi-annual periodicity, and residual short-term fluctuation components. These components are concatenated along the channel dimension to form a high-order temporal representation ${\text{H}}_{t}^{\text{S}}\in {\mathbb{R}}^{L\times N\times d}$. Finally, the outputs of the two branches are fused along the feature dimension, as shown in Formula 3:


$$\begin{array}{l} {\text{H}}_{t}=\text{Concat}\left({\text{H}}_{t}^{\text{G}},{\text{H}}_{t}^{\text{S}}\right), \end{array}$$ (3)

and then progressively mapped back to the spatial grid domain through a multilayer perceptron, a one-dimensional differentiable convolutional network, and a two-dimensional convolutional decoding head, yielding the predicted heatmap sequence for the next *H* months and enabling fine-grained spatio-temporal forecasting of HIV/AIDS case burden at provincial resolution.

### 2.4 Graph-aware spatio-temporal attention

**Figure 4:**
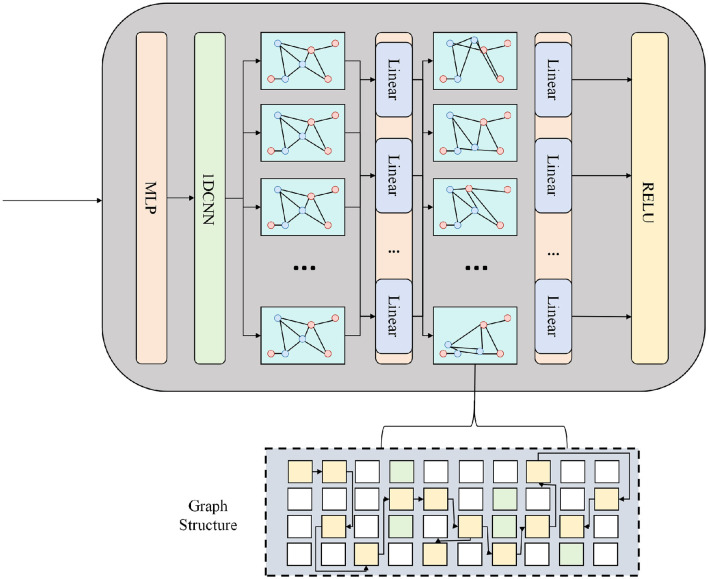
The diagram below illustrates the Graph-Aware Spatio-Temporal Attention module. The upper part shows the update process of the input provincial temporal features after sequentially passing through MLP, IDCNN, and multi-layer graph attention and linear transformation, thereby achieving joint modeling of spatial neighborhood and temporal dependence. The lower part shows the graph structure built based on the nodes of each province, used to characterize the topological connections between regions and to provide prior constraints for information propagation in graph attention.

In this section, the proposed graph-aware spatio-temporal attention module explicitly incorporates the provincial adjacency graph structure on top of the encoder outputs, enabling structure-constrained attention modeling over the case-count sequences. Let the features from the upstream 3D CNN and convolutional encoder be denoted as {**U**_*t*−*L*+1_, …, **U**_*t*_}, where the feature at each time step is ${\text{U}}_{\tau }\in {\mathbb{R}}^{N\times d_{\text{in}}}$, *N* is the number of provincial nodes, and *d*_in_ is the feature dimension. We first apply a shared multilayer perceptron to project node features into a unified latent space, yielding the initial spatio-temporal representations as shown in Formula 4:


$$\begin{array}{l} {\text{H}}_{\tau }^{\left(0\right)}=\phi \left({\text{U}}_{\tau }{\text{W}}_{\text{mlp}}+{\text{b}}_{\text{mlp}}\right),\;\tau =t-L+1,\ldots ,t, \end{array}$$ (4)

where ${\text{W}}_{\text{mlp}}\in {\mathbb{R}}^{d_{\text{in}}\times d}$ and ${\text{b}}_{\text{mlp}}\in {\mathbb{R}}^{d}$ are learnable parameters, *d* is the unified hidden dimension, and ϕ(·) is an element-wise nonlinearity. Here, ${\text{H}}_{\tau }^{\left(0\right)}\in {\mathbb{R}}^{N\times d}$ denotes the initial representation of each province at time step τ. Its module architecture is shown in Figure 4.

To capture short- and mid-range temporal dependencies prior to attention computation, we apply a one-dimensional dilated convolutional network to each node-wise time series. Let the time series of node *v*_*i*_ be ${\text{h}}_{i,t-L+1:t}^{\left(0\right)}\in {\mathbb{R}}^{L\times d}$, where *L* is the historical window length. Through multiple layers of dilated convolutions and nonlinear transformations, we obtain temporally smoothed features as shown in Formula 5:


$$\begin{array}{l} {\tilde{\text{h}}}_{i,t-L+1:t}=g_{\text{IDCNN}}\left({\text{h}}_{i,t-L+1:t}^{\left(0\right)}\right),\;i=1,\ldots ,N, \end{array}$$ (5)

where *g*_IDCNN_(·) denotes a stack of 1D convolutions with fixed dilation factors. The output ${\tilde{\text{h}}}_{i,t-L+1:t}\in {\mathbb{R}}^{L\times d}$ expands the receptive field while preserving temporal resolution and suppressing high-frequency noise. Aggregating the results across nodes yields the temporally modeled tensor ${\tilde{\text{H}}}_{\tau }^{\left(0\right)}\in {\mathbb{R}}^{N\times d}$.

Graph structural information is injected through a static undirected graph *G* = (*V, E, A*) constructed from provincial adjacency relations. In the adjacency matrix *A*∈{0, 1}^*N*×*N*^, *A*_*ij*_ = 1 indicates that provinces *v*_*i*_ and *v*_*j*_ share a boundary, and *A*_*ij*_ = 0 otherwise. Self-loops are added on the diagonal to form Â = *A*+**I**_*N*_. Let the degree matrix *D*∈ℝ^*N*×*N*^ be the diagonal matrix of row sums of Â. The normalized adjacency matrix is then defined as shown in Formula 6:


$$\begin{array}{l} Ã=D^{-\frac{1}{2}}ÂD^{-\frac{1}{2}}, \end{array}$$ (6)

where Ã_*ij*_ measures the relative structural proximity between nodes *v*_*i*_ and *v*_*j*_. Based on Ã, the module further learns a static structural embedding for each node as shown in Formula 7:


$$\begin{array}{l} \text{S}=Ã{\text{W}}_{g}, \end{array}$$ (7)

where ${\text{W}}_{g}\in {\mathbb{R}}^{N\times d_{g}}$ is a learnable parameter matrix. The row vector **s**_*i*_ of $\text{S}\in {\mathbb{R}}^{N\times d_{g}}$ characterizes the position and neighborhood pattern of node *v*_*i*_ in the global graph structure.

At the ℓ-th layer of the graph-aware multi-head attention block, we take ${\tilde{\text{H}}}_{\tau }^{\left(\ell \right)}$ as input and perform node-wise self-attention independently at each time step. For the *h*-th attention head, we first apply linear projections to obtain the query, key, and value matrices as shown in Formula 8:


$$\begin{array}{l} {\text{Q}}_{\tau }^{\left(h\right)}={\tilde{\text{H}}}_{\tau }^{\left(\ell \right)}{\text{W}}_{Q}^{\left(h\right)},\;{\text{K}}_{\tau }^{\left(h\right)}={\tilde{\text{H}}}_{\tau }^{\left(\ell \right)}{\text{W}}_{K}^{\left(h\right)},\;{\text{V}}_{\tau }^{\left(h\right)}={\tilde{\text{H}}}_{\tau }^{\left(\ell \right)}{\text{W}}_{V}^{\left(h\right)}, \end{array}$$ (8)

where ${\text{W}}_{Q}^{\left(h\right)},{\text{W}}_{K}^{\left(h\right)},{\text{W}}_{V}^{\left(h\right)}\in {\mathbb{R}}^{d\times d_{k}}$ are the projection matrices for head *h*. Accordingly, ${\text{Q}}_{\tau }^{\left(h\right)},{\text{K}}_{\tau }^{\left(h\right)},{\text{V}}_{\tau }^{\left(h\right)}\in {\mathbb{R}}^{N\times d_{k}}$, whose row vectors represent node embeddings in the query, key, and value spaces, respectively. To explicitly integrate structural embeddings into attention weights, we introduce a bias matrix *B*∈ℝ^*N*×*N*^ constructed from node structural embeddings, with elements defined as $B_{ij}={\text{s}}_{i}^{⊤}{\text{s}}_{j}$, and we constrain the attention scope using the normalized adjacency matrix.

Combining feature similarity and graph-structured constraints, the inter-node attention weight for head *h* at time step τ is defined as shown in Formula 9:


$$\begin{array}{l} \alpha_{ij,\tau }^{\left(h\right)}=\frac{\exp\left({Ã}_{ij}\frac{{\text{q}}_{i,\tau }^{\left(h\right)}{\text{k}}_{j,\tau }^{\left(h\right)⊤}}{\sqrt{d_{k}}}+\beta B_{ij}\right)}{\overset{N}{\underset{k=1}{\sum }}\exp\left({Ã}_{ik}\frac{{\text{q}}_{i,\tau }^{\left(h\right)}{\text{k}}_{k,\tau }^{\left(h\right)⊤}}{\sqrt{d_{k}}}+\beta B_{ik}\right)}, \end{array}$$ (9)

where ${\text{q}}_{i,\tau }^{\left(h\right)}$ and ${\text{k}}_{j,\tau }^{\left(h\right)}$ are the *i*-th and *j*-th row vectors of ${\text{Q}}_{\tau }^{\left(h\right)}$ and ${\text{K}}_{\tau }^{\left(h\right)}$, respectively, and β is a scalar hyperparameter controlling the strength of the structural bias. The coefficient $\alpha_{ij,\tau }^{\left(h\right)}$ represents the weight with which node *v*_*i*_ aggregates information from node *v*_*j*_ at time step τ under the graph constraint. Using these weights, we compute the weighted sum ${\text{z}}_{i,\tau }^{\left(h\right)}=\overset{N}{\underset{j=1}{\sum }}\alpha_{ij,\tau }^{\left(h\right)}{\text{v}}_{j,\tau }^{\left(h\right)}$, and then concatenate the outputs across all heads followed by an output projection to obtain the node features at layer ℓ+1 as shown in Formula 10:


$$\begin{array}{l} {\tilde{\text{H}}}_{\tau }^{\left(\ell +1\right)}=\sigma \left(\text{Conca}{\text{t}}_{h=1}^{H_{\text{head}}}\left[{\text{Z}}_{\tau }^{\left(h\right)}\right]{\text{W}}_{O}+{\tilde{\text{H}}}_{\tau }^{\left(\ell \right)}\right), \end{array}$$ (10)

where ${\text{Z}}_{\tau }^{\left(h\right)}\in {\mathbb{R}}^{N\times d_{k}}$ consists of all ${\text{z}}_{i,\tau }^{\left(h\right)}$, *H*_head_ is the number of attention heads, and ${\text{W}}_{O}\in {\mathbb{R}}^{H_{\text{head}}d_{k}\times d}$ is the output projection matrix. The element-wise nonlinearity σ(·) and the residual term ${\tilde{\text{H}}}_{\tau }^{\left(\ell \right)}$ stabilize the fusion between the input features and graph-aware updates. After stacking multiple layers, the resulting spatio-temporal feature sequence encodes both the temporal evolution of cases and the provincial adjacency structure at the node level, providing high-quality graph-aware representations for the loss function.

### 2.5 Seasonal structure-aware temporal module

**Figure 5:**
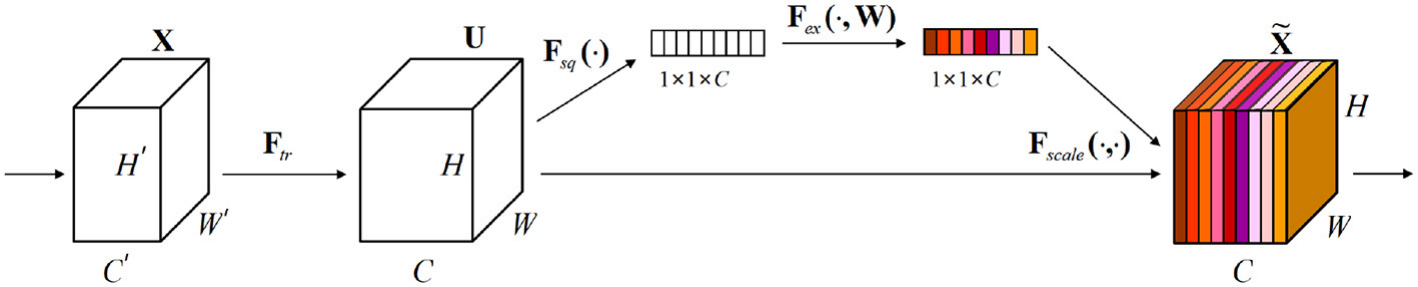
3DCNN model architecture diagram.

In the seasonal structure-aware temporal module, we explicitly model the trend and seasonal components of the HIV/AIDS case sequence on top of the spatio-temporal features extracted by the 3DCNN encoder. Let the feature at time step *t* after the 3DCNN and the main encoder be ${\text{H}}_{t}\in {\mathbb{R}}^{N\times D}$, where *N* denotes the number of provincial administrative units and *D* is the latent dimension for each node. We first pool along the spatial (node) dimension to compress node-level representations into a temporal feature vector:


$$\begin{array}{l} {\text{z}}_{t}=\text{Pool}\left({\text{H}}_{t}\right)\in {\mathbb{R}}^{D},\;t=1,\ldots ,T, \end{array}$$ (11)

as shown in Formula 11, where Pool(·) denotes an average-pooling or weighted-pooling operator over the node dimension, and *T* is the sequence length. Stacking *L* consecutive time steps yields the input matrix


$$\begin{array}{l} \text{Z}=[{\text{z}}_{t-L+1},\ldots ,{\text{z}}_{t}{]}^{⊤}\in {\mathbb{R}}^{L\times D}, \end{array}$$ (12)

as shown in Formula 12, which serves as the deterministic input to the seasonal structure-aware temporal module and preserves the global temporal evolution encoded by the 3DCNN. The model architecture of 3DCNN is shown in Figure 5.

To extract stable seasonal components from **Z**, we adopt a stack of one-dimensional convolutional layers to perform local modeling along the temporal dimension. Let the parameters of the *l*-th convolution layer be a weight tensor ${\text{W}}^{\left(l\right)}\in {\mathbb{R}}^{K_{l}\times D\times D}$ and a bias vector **b**^(*l*)^∈ℝ^*D*^, where *K*_*l*_ is the kernel size. The output of the *l*-th layer, **S**^(*l*)^∈ℝ^*L*×*D*^, is defined as


$$\begin{array}{l} {\text{S}}^{\left(l\right)}=\sigma \left(\text{Conv}1\text{D}\left({\text{S}}^{\left(l-1\right)};{\text{W}}^{\left(l\right)}\right)+{\text{b}}^{\left(l\right)}\right),\;l=1,\ldots ,L_{s}, \end{array}$$ (13)

as shown in Formula 13, where **S**^(0)^ = **Z**, σ(·) is an element-wise nonlinear activation, and *L*_*s*_ denotes the number of convolution layers. This procedure smooths and reconstructs the case dynamics within a fixed temporal window, so that the deep output ${\text{S}}^{\left(L_{s}\right)}$ primarily captures periodic oscillations while suppressing high-frequency noise. To preserve the slowly varying trend component, we further construct a trend feature matrix:


$$\begin{array}{l} \text{T}=\text{AvgPool}\left(\text{Z}\right)1_{L}^{⊤}\in {\mathbb{R}}^{L\times D}, \end{array}$$ (14)

as shown in Formula 14, where AvgPool(**Z**)∈ℝ^1 × *D*^ is the average pooling result over the temporal dimension and $1_{L}\in {\mathbb{R}}^{L}$ is an all-ones column vector, yielding a smoothed trend baseline **T** with the same shape as **Z**.

To more finely characterize seasonal structures across different temporal scales, we introduce multi-dilation convolutional channels on top of ${\text{S}}^{\left(L_{s}\right)}$. Let the predefined dilation set be {*d*_1_, …, *d*_*M*_}, with corresponding kernel weights ${\text{W}}_{m}^{\text{sea}}\in {\mathbb{R}}^{K_{m}\times D\times D}$. The output of the *m*-th seasonal channel is


$$\begin{array}{l} {\text{S}}_{m}=\text{Conv}1\text{D}\left({\text{S}}^{\left(L_{s}\right)};{\text{W}}_{m}^{\text{sea}},d_{m}\right)\in {\mathbb{R}}^{L\times D},\;m=1,\ldots ,M, \end{array}$$ (15)

as shown in Formula 15, where *d*_*m*_ is the dilation rate along the temporal dimension. Convolutions with different *d*_*m*_ explicitly align with periodic structures of length *d*_*m*_ in monthly case counts, such as annual or quarterly cycles. We then fuse the multi-scale seasonal channels via a weighted aggregation to obtain the final seasonal representation:


$$\begin{array}{l} \hat{\text{S}}=\overset{M}{\underset{m=1}{\sum }}\alpha_{m}{\text{S}}_{m}\in {\mathbb{R}}^{L\times D}, \end{array}$$ (16)

as shown in Formula 16, where α_*m*_∈ℝ is a learnable scalar weight satisfying $\overset{M}{\underset{m=1}{\sum }}\alpha_{m}=1$, controlling the contribution of each time-scale seasonal component to the overall representation.

Given the trend representation **T** and the seasonal representation $\hat{\text{S}}$, we construct prediction-oriented temporal features via a gated fusion mechanism. Let the gating parameters be ${\text{W}}_{g}\in {\mathbb{R}}^{2D\times D}$ and ${\text{b}}_{g}\in {\mathbb{R}}^{D}$. For each time step *i* = 1, …, *L*, the gate vector ${\text{g}}_{i}\in {\mathbb{R}}^{D}$ is defined as


$$\begin{array}{l} {\text{g}}_{i}=\sigma \left(\left[{\text{T}}_{i},{\hat{\text{S}}}_{i}\right]{\text{W}}_{g}+{\text{b}}_{g}\right), \end{array}$$ (17)

as shown in Formula 17, where ${\text{T}}_{i},{\hat{\text{S}}}_{i}\in {\mathbb{R}}^{D}$ are the *i*-th row vectors of the trend and seasonal features, respectively, and [·, ·] denotes concatenation along the feature dimension. Based on this gate, the final temporal representation is computed as


$$\begin{array}{l} {\text{u}}_{i}={\text{g}}_{i}⊙{\hat{\text{S}}}_{i}+\left(1-{\text{g}}_{i}\right)⊙{\text{T}}_{i}\in {\mathbb{R}}^{D}, \end{array}$$ (18)

as shown in Formula 18, where ⊙ denotes element-wise multiplication. The vector **u**_*i*_ is the deterministic output of the seasonal structure-aware temporal module at time step *i*, which is subsequently fused with the spatial features from the 3DCNN–Encoder branch and the Graph-Aware Spatio-Temporal Attention branch during decoding to construct the case-count prediction function.

## 3 Results

### 3.1 Experimental setup

All experiments in this study were conducted under the same computational environment to ensure that results are comparable across different models and configurations. The hardware platform consisted of a single NVIDIA RTX 3090 GPU (24 GB memory), a 16-core CPU (Intel Core i9-12900K), and 128 GB of physical RAM, running Ubuntu 20.04. In terms of software, Python 3.10 was used as the development language, with PyTorch 2.2 as the deep learning framework, CUDA 11.8 and the corresponding cuDNN installed for GPU acceleration. Data processing and visualization primarily relied on libraries including pandas, NumPy, GeoPandas, and Matplotlib. All experiments were executed independently in this environment without multi-node or multi-GPU training to ensure stable and reproducible evaluation.

For hyperparameter settings, the historical window length was set to *L* = 12 months and the forecasting horizon to *H* = 1 month. The data were split chronologically into 60% for training, 20% for validation, and 20% for testing to avoid information leakage. The batch size was fixed at 32. We used AdamW as the optimizer with an initial learning rate of 1 × 10^−3^ and a weight decay of 1 × 10^−4^. A cosine annealing learning-rate scheduler with warmup was adopted, using 5 warmup epochs and a total of 200 training epochs. The 3DCNN encoder contained two 3D convolution layers with channel sizes 32 and 64, respectively, and a kernel size of 3 × 3 × 3. In the Graph-Aware Spatio-Temporal Attention branch, the graph embedding dimension was set to 64 and the number of spatio-temporal attention layers to 2. In the Seasonal Structure-Aware Temporal Module, the number of 1D convolution layers was set to 3 with the dilation set {1, 3, 6}, and the dropout rate for all linear and convolution layers was fixed at 0.1. A summary of the main experimental settings is provided in Table 1.

**Table 1:** Summary of the hardware environment and key hyperparameter settings.

**Category**	**Item**	**Setting**
Hardware	GPU	NVIDIA RTX 3090, 24 GB memory
	CPU	Intel Core i9-12900K, 16 cores
	Memory	128 GB RAM
	Operating system	Ubuntu 20.04
Software	Python version	3.10
	Deep learning framework	PyTorch 2.2
	CUDA/cuDNN	CUDA 11.8 with cuDNN
	Data and plotting libraries	Pandas, NumPy, GeoPandas, Matplotlib
Training setup	Window length *L*	12 months
	Forecast horizon *H*	1 month
	Data split	60%/20%/20% (train/val/test)
	Batch size	32
	Epochs	200
Optimizer	Type	AdamW
	Initial learning rate	1 × 10^−3^
	Weight decay	1 × 10^−4^
	LR schedule	5-epoch warmup + cosine annealing
Model design	3DCNN channels	32 → 64
	3DCNN kernel	3 × 3 × 3
	Graph embedding dimension	64
	Spatio-temporal attention layers	2
	Seasonal conv layers	3
	Dilation set	{1, 3, 6}
	Dropout	0.1

### 3.2 Evaluation metric

To comprehensively evaluate the model performance for spatio-temporal forecasting of HIV/AIDS case counts, this study adopts four metrics—Mean Squared Error (MSE), Mean Absolute Error (MAE), Peak Signal-to-Noise Ratio (PSNR), and Structural Similarity Index Measure (SSIM)—to quantify both the numerical deviation and the perceptual quality between the predicted and ground-truth case distributions. Let *y*_*i*_ denote the true case count for a given time and region, ŷ_*i*_ the corresponding prediction, and *N* the total number of samples.

First, MSE measures the overall squared error magnitude on the numerical scale as shown in Formula 19:


$$\begin{array}{l} \text{MSE}=\frac{1}{N}\overset{N}{\underset{i=1}{\sum }}{\left({ŷ}_{i}-y_{i}\right)}^{2}. \end{array}$$ (19)

This metric penalizes large errors more heavily and is therefore sensitive to severe overestimation or underestimation of case counts; a smaller MSE indicates more accurate predictions.

Second, MAE focuses on the mean absolute deviation and characterizes the overall magnitude of numerical bias as shown in Formula 20:


$$\begin{array}{l} \text{MAE}=\frac{1}{N}\overset{N}{\underset{i=1}{\sum }}\left|{ŷ}_{i}-y_{i}\right|. \end{array}$$ (20)

Compared with MSE, MAE is less sensitive to outliers and more directly reflects the average prediction error at each spatial unit and time point; a smaller MAE implies more stable numerical fitting.

When comparing heatmaps at the image level, PSNR is used to measure the signal-to-noise ratio between the predicted intensity map and the ground-truth map as shown in Formula 21:


$$\begin{array}{l} \text{PSNR}=10\log_{10}\left(\frac{\text{MA}{\text{X}}^{2}}{\text{MSE}}\right), \end{array}$$ (21)

where MAX denotes the maximum pixel value of the normalized intensity map (in this study, MAX = 1). A higher PSNR indicates smaller overall distortion between the predicted and true maps in the visualization space. In epidemiological surveillance, this distortion is interpreted as the degree to which the model preserves the overall spatial intensity field after rasterization, i.e., whether the predicted risk surface deviates substantially from the observed distribution at the map level rather than only at isolated provinces.

Finally, SSIM evaluates the perceptual similarity between the predicted heatmap and the ground-truth heatmap in terms of luminance, contrast, and structural consistency as shown in Formula 22:


$$\begin{array}{l} \text{SSIM}\left(x,y\right)=\frac{\left(2\mu_{x}\mu_{y}+C_{1}\right)\left(2\sigma_{xy}+C_{2}\right)}{\left(\mu_{x}^{2}+\mu_{y}^{2}+C_{1}\right)\left(\sigma_{x}^{2}+\sigma_{y}^{2}+C_{2}\right)}, \end{array}$$ (22)

where *x* and *y* denote the sets of pixels within a local window from the predicted and ground-truth maps, respectively; μ_*x*_ and μ_*y*_ are local means, $\sigma_{x}^{2}$ and $\sigma_{y}^{2}$ are local variances, σ_*xy*_ is the covariance, and *C*_1_ and *C*_2_ are stabilizing constants to avoid division by zero. The SSIM value ranges from 0 to 1, with larger values indicating that the prediction is more consistent with the true epidemic pattern in terms of spatial structural characteristics. We employ SSIM as a complementary surrogate for actionable pattern consistency, because public-health decisions often rely on the relative spatial configuration of high-burden areas (e.g., hotspot extent and spatial gradients) in addition to exact numeric counts; thus, a higher SSIM suggests better preservation of the spatial structure that underpins hotspot-oriented surveillance.

### 3.3 Comparison of experimental results with other models

To systematically evaluate the overall performance of the proposed Graph-Aware Spatio-Temporal Attention and Seasonal Structure-Aware Temporal Module for HIV/AIDS case count forecasting, this section selects a set of representative spatio-temporal prediction models as comparative baselines, covering classical convolution–recurrent structures, spatio-temporal networks based on vector-field modeling, diffusion-style temporal generative models, and recent Transformer-family architectures designed for urban and public health scenarios. All models were re-implemented or reproduced under the same data splits, evaluation metrics, and training protocol. We report quantitative comparisons using four metrics—Mean Squared Error (MSE), Mean Absolute Error (MAE), Structural Similarity Index Measure (SSIM), and Peak Signal-to-Noise Ratio (PSNR)—thereby assessing the relative strengths and complementarities of different methods for HIV/AIDS spatio-temporal forecasting from two perspectives: numerical fitting accuracy and spatial-structural fidelity. The experimental results are summarized in Table 2.

**Table 2:** Comparison with baseline models on HIV/AIDS case-count forecasting.

**Method**	**MSE**	**MAE**	**SSIM**	**PSNR**
Unist (23)	21.8427	2.3174	0.812	29.41
Diffirm (24)	18.5623	2.0041	0.834	30.27
Mgsfformer (25)	15.4379	1.7625	0.856	31.12
Urbangpt (26)	14.9821	1.7033	0.861	31.38
SimVPv2 (27)	14.5574	1.6649	0.867	31.59
Vmrnn (28)	13.8745	1.5832	0.872	31.90
Bayesian ST model (29)	21.3752	2.2416	0.801	29.08
INLA-based NB model (30)	24.6758	2.5039	0.778	28.32
Ours	12.5336	1.4546	0.889	32.74

From Table 2, it can be observed that compared with a range of representative spatio-temporal forecasting baselines, our method achieves the lowest values on both MSE and MAE, while attaining the highest scores on SSIM and PSNR. This indicates clear advantages in terms of both numerical accuracy and spatial-structural fidelity. Relative to convolution- or recurrent-based models such as SimVPv2 and Vmrnn, the further performance gains suggest that relying solely on local convolutions or sequential modeling is insufficient to fully capture the complex inter-provincial spatial dependencies present in HIV/AIDS case dynamics. By explicitly incorporating graph-structured adjacency relations into spatio-temporal attention weighting, the Graph-Aware Spatio-Temporal Attention component enables the model to assign larger weights to highly correlated provinces and critical propagation pathways during forecasting, thereby achieving finer-grained control over overall prediction errors. The particularly strong improvement in SSIM further implies that this attention mechanism better preserves the spatial morphology of epidemic patterns than baselines that depend on fixed convolution kernels or rigid neighborhood definitions.

In addition, our method consistently outperforms Transformer-centric baselines such as Mgsfformer and Urbangpt, indicating that self-attention alone may be inadequate for capturing seasonality and multi-scale temporal dependencies in HIV/AIDS epidemic processes. The Seasonal Structure-Aware Temporal Module decomposes the case time series into trend, seasonal, and residual components, and, together with 3DCNN-based convolutional encoding over multi-scale spatio-temporal blocks, allows the model to explicitly and selectively model both long-term background evolution and stable seasonal fluctuations. This design reduces the burden on attention layers when learning long sequences, while injecting seasonal-structure information into temporal representations in an explicit manner, leading to smoother and more stable forecasts as reflected by improvements in MAE and PSNR. Taken together, the consistent gains across all four metrics suggest that the graph-aware spatio-temporal attention mechanism and the seasonal structure-aware temporal module are complementary in this task, substantially enhancing the model's capacity to characterize complex spatio-temporal propagation patterns of HIV/AIDS.

### 3.4 Ablation experimental results

Although the overall model achieves strong forecasting performance, it remains necessary to further analyze the marginal contributions and underlying mechanisms of each key component in improving HIV/AIDS spatio-temporal case-count prediction. To this end, this section conducts a series of ablation studies focusing on the Graph-Aware Spatio-Temporal Attention module, the Seasonal Structure-Aware Temporal Module, and the multi-scale spatio-temporal encoding achieved through the integration with 3DCNN. Specifically, we progressively remove or simplify individual components and compare the resulting variants under the same experimental settings. By examining how the evaluation metrics change across different configurations, we can more clearly characterize the independent value and synergistic gains of each module in capturing inter-provincial propagation dependencies, seasonal structures, and high-dimensional spatio-temporal patterns. The ablation results are summarized in Table 3.

**Table 3:** Ablation study of the proposed model components.

**Method**	**MSE**	**MAE**	**SSIM**	**PSNR**
3DCNN (baseline)	18.7421	2.0835	0.842	30.12
+ GASTA	16.3849	1.8732	0.859	30.97
+ SSATM	14.2734	1.6727	0.874	31.68
Ours	12.5336	1.4546	0.889	32.74

As shown in Table 3, under the same data split and training configuration, the plain 3DCNN baseline performs the worst across all four metrics, indicating that relying solely on local convolutions provides limited capacity for modeling the complex spatio-temporal dependencies of HIV/AIDS case dynamics. After introducing the Graph-Aware Spatio-Temporal Attention module (GASTA), both MSE and MAE decrease noticeably, while SSIM and PSNR improve steadily, suggesting that explicitly leveraging the inter-provincial graph structure together with spatio-temporal attention weights enables more effective capture of propagation pathways and cross-region correlation patterns. Building on this, adding the Seasonal Structure-Aware Temporal Module (SSATM) further induces an explicit decomposition-oriented representation of long-term trends and seasonal cycles, leading to consistent additional gains in both numerical errors and structural similarity. Finally, the full model that integrates GASTA, SSATM, and the multi-scale encoding capability of 3DCNN achieves the best results on MSE, MAE, SSIM, and PSNR, verifying the complementarity and synergistic benefits of graph-aware spatio-temporal attention and seasonal structure-aware temporal modeling for HIV/AIDS case count forecasting.

### 3.5 Spatio-temporal error distribution across regions

**Figure 6:**
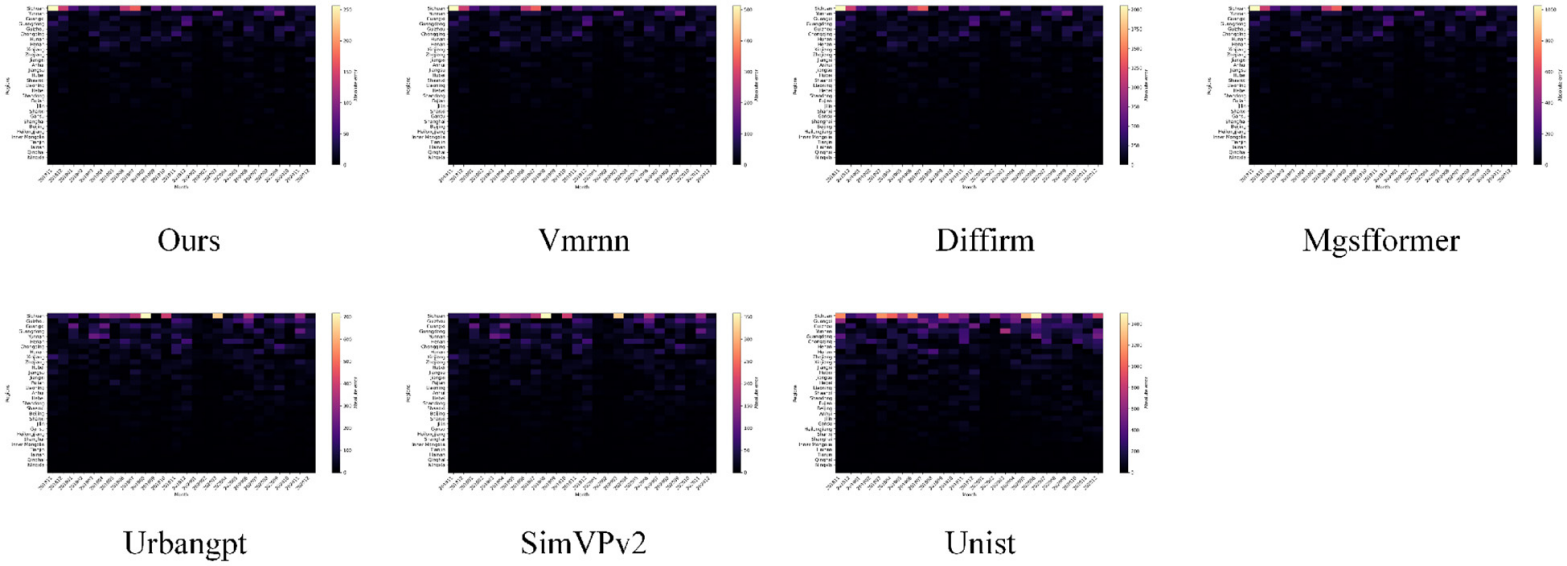
Month–region heatmaps of pointwise absolute error on the validation set for different forecasting methods, where brighter colors indicate larger deviations from the ground truth.

To further analyze the bias structure of HIV/AIDS case-count forecasting from a spatio-temporal perspective, this section visualizes the pointwise absolute errors of different methods on a two-dimensional “month–region” grid. Specifically, we construct an error matrix by aggregating the absolute prediction errors for all provincial-level administrative units across all months in the validation period, and then render the matrix as a heatmap to characterize how each method distributes errors across high-burden and low-burden regions. This visualization provides an overall view of the joint evolution of forecasting errors over time and space, offering an intuitive basis for subsequent regional disparity analyses and model diagnostics. The corresponding results are shown in Figure 6.

As shown in the figure, the month–region absolute error heatmap of our method exhibits an overall darker color scale, indicating smaller forecasting deviations for HIV/AIDS case counts across most provinces and months. This suggests a more uniform and stable spatio-temporal error distribution, which is beneficial for continuous surveillance and resource allocation by public health authorities. In contrast, methods such as Diffirm and Mgsfformer display pronounced bright bands in certain months within high-burden provinces, implying systematic underestimation or overestimation during localized outbreaks or abrupt structural changes in case patterns, which may weaken early-warning capability for priority areas. Models that primarily rely on temporal sequence modeling, such as Vmrnn and SimVPv2, also present scattered bright spots in some coastal and central–western regions, reflecting insufficient characterization of cross-regional transmission dependencies and spatial heterogeneity. By explicitly incorporating the inter-provincial graph structure to represent potential propagation channels via Graph-Aware Spatio-Temporal Attention, and by decomposing long-term trends and seasonal cycles through the Seasonal Structure-Aware Temporal Module, our approach maintains lower errors in both high-prevalence regions and critical seasons, thereby providing a more reliable spatio-temporal risk characterization to support public health decisions such as zonal interventions and graded deployment of prevention and control resources.

### 3.6 Point-wise consistency between predictions and observations

**Figure 7:**
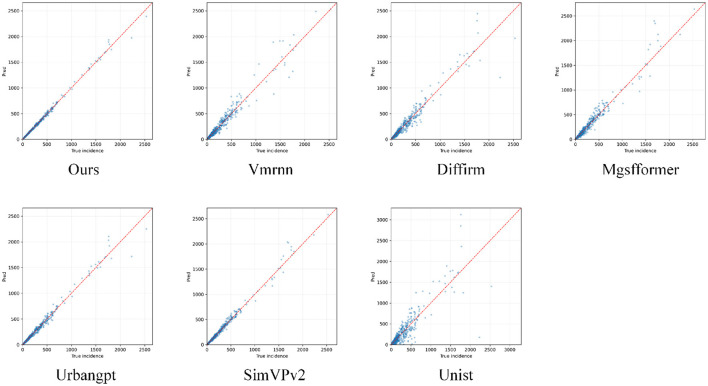
Scatter plots of predicted vs. observed HIV/AIDS case counts on the validation set for different forecasting methods. Each sub-figure corresponds to one model and visualizes province–month pairs as points in the “true incidence–predicted incidence” space, with the red dashed line indicating the ideal unbiased reference *y* = *x*.

To further evaluate the overall numerical fitting capability of the model, this section constructs a scatter plot of ground-truth vs. predicted case counts for all “province–month” samples in the validation set, enabling pointwise comparison between the two. This experiment provides an intuitive assessment of the model's consistency across different burden levels and helps reveal potential systematic biases, thereby establishing a basis for subsequent analyses of error distributions and robustness. The corresponding results are shown in Figure 7.

From the scatter plot, clear discrepancies can be observed between the predicted and ground-truth HIV/AIDS case counts at the province–month level across different methods. The point cloud of our approach is overall more tightly clustered around the reference diagonal line, indicating that case-scale estimates are closer to the true epidemic situation across different burden levels. In contrast, several competing models exhibit systematic deviations in the high case-count range, where peak values in high-burden provinces are often under- or over-estimated; such bias may lead to misallocation of prevention and control resources in priority regions. For low-prevalence areas, some methods show overly dispersed and fluctuating scatter distributions in sporadic months, suggesting substantial uncertainty in identifying emerging hotspots, which is unfavorable for early warning and fine-grained interventions. The more diagonal-aligned point distribution achieved by our method implies more stable characterization of case magnitudes across regions and time periods, providing a more reliable quantitative epidemiological basis for designing tiered prevention strategies and targeted population-level interventions.

### 3.7 The difference between the actual and predicted values for all provinces as a whole

**Figure 8:**
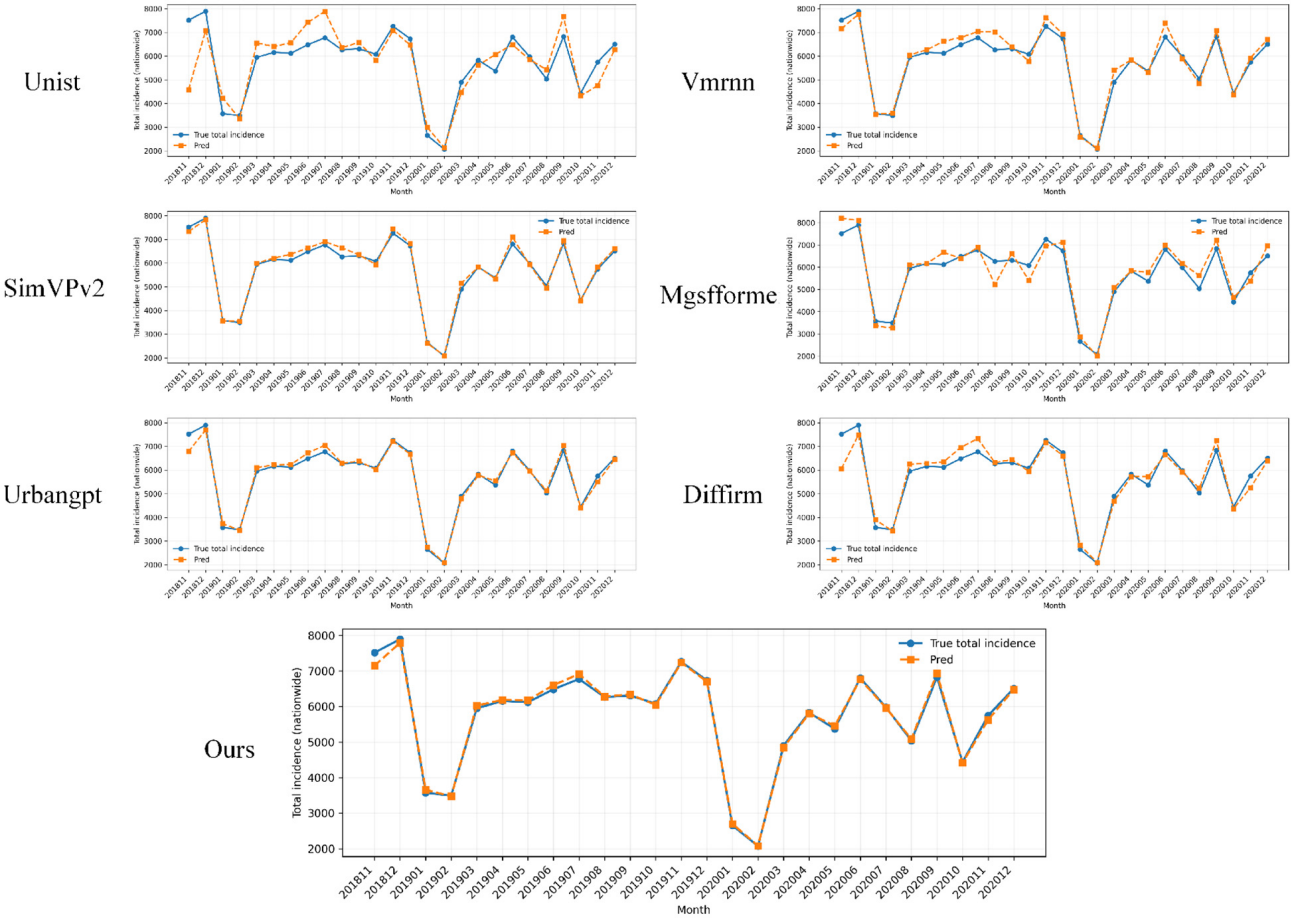
Comparison of national monthly HIV/AIDS case-count trajectories on the validation set, where each subplot corresponds to one forecasting model and plots true total incidence and predicted total incidence over time. The curves for different months provide a holistic view of how each method captures temporal variations in aggregate case counts at the country level.

After comparing the overall performance of various baseline models and the proposed method, it is necessary to systematically examine the bias structure between predictions and observed case counts at the national scale. To this end, this subsection adopts a global perspective across all provinces and time points and conducts a pointwise comparison between model outputs and observed cases, aiming to reveal the overall error distribution and potential systematic bias patterns at the nationwide level. The corresponding results are shown in Figure 8.

As shown in the figure, all methods can roughly track the long-term fluctuations of the national monthly HIV/AIDS case totals, yet their fitting accuracy differs substantially around peaks and troughs. Several baseline models exhibit noticeable lag or overshooting during months with rapid increases or sharp declines, leading to systematic underestimation or overestimation of case magnitudes in high-burden periods, which may introduce risks for public health resource deployment and peak-stage early warning. In contrast, the proposed model maintains a closer trajectory alignment near multiple pronounced epidemic crests and bottoms, suggesting that Graph-Aware Spatio-Temporal Attention together with seasonal-structure modeling better captures inter-provincial propagation effects and periodic dynamics. From a practical public health perspective, such more stable and lower-bias forecasting at the national aggregate level can support more accurate anticipation of future clinical demand and medication needs, providing a more reliable quantitative basis for formulating national prevention and control strategies.

### 3.8 The algorithm and baseline presented in this paper were tested in provinces with the highest number of cases

**Figure 9:**
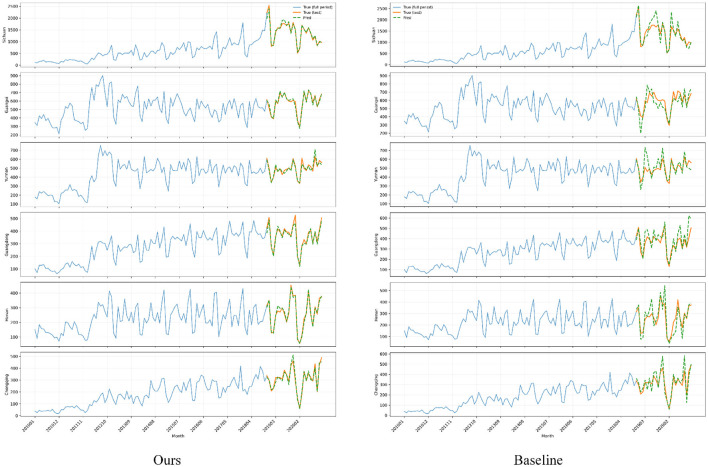
Province-level time series of monthly HIV/AIDS case counts in several high-burden regions, where each row corresponds to one province and the left and right columns show the forecasts from the proposed model and a baseline model, respectively. For each panel, the long blue curve denotes the historical incidence over the entire study period, and the colored segments at the end mark the ground-truth and predicted values on the validation horizon.

In addition to nationwide analyses, the proposed algorithm and all baseline models are further examined on provinces with the highest cumulative HIV/AIDS case counts over the study period. Focusing on these high-burden regions allows us to assess whether the forecasting methods can faithfully capture epidemic dynamics precisely where accurate surveillance is most critical for public health planning and resource allocation. The experimental results are shown in Figure 9.

From the provincial-level time-series comparisons, it can be observed that in several regions with the highest case burdens, the predicted curves produced by the model generally follow the oscillation rhythm of the historical incidence trajectories. Notably, the predictions remain highly consistent even during rapid increases and short-term fluctuations, which is crucial for early identification of localized outbreaks and periods of tightened resource capacity. In contrast, baseline models tend to exhibit lag or over-reactive behavior around high-incidence seasons and peak phases in certain provinces, causing noticeable deviations between predicted and observed curves at key turning points and thereby weakening the ability to identify effective windows for public health intervention. The smoother yet closely aligned predicted trajectories indicate that, after incorporating graph-aware spatio-temporal attention and seasonal structure-aware temporal modeling, the model can simultaneously capture long-term upward trends and seasonal cycles, while allocating risk weights more precisely along cross-provincial transmission chains. For real-world surveillance systems, this ability to maintain stable accuracy in priority provinces and critical periods supports tiered early warning and graded allocation of testing and treatment resources, improving overall prevention and control efficiency under constrained public health budgets.

### 3.9 Model stability experimental results

To further validate the usability and reproducibility of the proposed method in practical public health surveillance settings, we conduct a stability analysis on key structural hyperparameters, with a particular focus on how the dimensionality of the graph representation space affects overall forecasting behavior. Specifically, the graph embedding dimension directly determines the representational capacity and compression strength of inter-provincial relational information in the latent space, thereby influencing the modeling accuracy of cross-regional propagation pathways within the graph-aware spatio-temporal attention module, as well as the consistency of its subsequent fusion with seasonal structure-aware temporal representations. By systematically varying this dimension under the same data split and training configuration, we can assess the robustness boundary and sensitivity characteristics of the model across different capacity regimes, and provide actionable guidance for hyperparameter selection when deploying the framework to regions of different scale and surveillance granularity. The experimental results are summarized in Table 4.

**Table 4:** Sensitivity analysis of graph embedding dimension.

**Graph embedding dimension**	**MSE**	**MAE**	**SSIM**	**PSNR**
256	13.1024	1.4921	0.852	31.98
128	12.8745	1.4683	0.881	32.41
32	14.2279	1.5714	0.861	31.23
64	12.5336	1.4546	0.889	32.74

This sensitivity study indicates that the graph embedding dimension has a pronounced impact on both forecasting accuracy and structural consistency. When the dimension is too small, the representational capacity becomes insufficient to adequately encode inter-provincial propagation relationships and cross-month dependencies, leading to increased numerical errors and concurrent decreases in SSIM and PSNR. As the dimension increases to a moderate scale, graph-structured information can be injected into spatio-temporal attention and seasonal-structure modeling in a richer and more robust manner, yielding further convergence of error metrics and improvements in structural similarity. This suggests stronger discriminability in capturing fluctuations in high-burden provinces and changes during critical seasons. However, when the dimension is further increased to 256, performance deteriorates, implying that overly large embeddings may introduce redundant degrees of freedom and amplify sensitivity to noise, making the fusion between graph representations and temporal modules harder to stabilize and thus less favorable for generalization in public health surveillance settings where signals are limited and noise is complex.

### 3.10 Multi-step prediction experiment results

To enhance the translational relevance of the proposed framework for public health surveillance, we further extend the evaluation from the 1-month-ahead setting to multi-step forecasting over longer horizons. Specifically, given an observation window of length *L*, the model is required to generate a sequence of future provincial case-count heatmaps $\left\{{\hat{\text{I}}}_{t+1},{\hat{\text{I}}}_{t+2},\ldots ,{\hat{\text{I}}}_{t+H}\right\}$, where *H* denotes the forecasting horizon. This setting better reflects practical needs such as staffing, programme planning and supply management, and allows us to examine how predictive accuracy and spatial pattern consistency evolve as the forecast horizon increases. The experimental results are shown in Table 5.

**Table 5:** Multi-step prediction performance under different forecasting horizons.

**Step**	**MSE**	**MAE**	**SSIM**	**PSNR**
1	12.5336	1.4546	0.889	32.74
3	17.9421	1.9623	0.852	30.44
6	24.8065	2.5317	0.771	27.98

As the forecasting horizon increases, the numerical and structural fidelity of predictions degrades in a consistent manner, reflecting the accumulation of uncertainty and the growing difficulty of maintaining accurate spatio-temporal dynamics over longer lead times. Compared with the one-step setting, both MSE and MAE rise substantially at 3-step and 6-step horizons, indicating enlarged absolute deviations and more frequent larger-magnitude errors in provincial case counts. Meanwhile, SSIM and PSNR decrease monotonically from 0.889/32.74 to 0.771/27.98, suggesting that the predicted heatmaps progressively lose fine-grained spatial structure and exhibit greater overall distortion relative to the ground-truth intensity fields, which is expected when multi-step forecasts must extrapolate evolving epidemic patterns further into the future.

### 3.11 Public health indicator analysis

Beyond image-level similarity and overall numerical errors, public health surveillance requires model outputs to be interpretable in terms of actionable epidemiological signals, such as month-to-month changes in burden and the identification of high-risk provinces that warrant priority attention. In this subsection, we therefore introduce additional indicator-oriented analyses that evaluate whether the proposed framework preserves the temporal direction and magnitude of changes across provinces and maintains consistent hotspot ranking patterns over time, thereby better aligning the assessment with practical decision-making needs in prevention planning and resource allocation. The experimental results are shown in Table 6.

**Table 6:** Public health indicator analysis based on relative-change error and hotspot ranking consistency.

**Category**	**Metric**	**Value**
Relative-change error (Δ*y* = *y*_*t*_−*y*_*t*−1_)	Δ*y* MAE (all provinces)	2.6894
	Δ*y* RMSE (all provinces)	5.0440
	Δ*y* MAE (high-burden)	2.8540
	Δ*y* RMSE (high-burden)	5.2515
Hotspot ranking consistency (per month)	Spearman ρ (mean ± std)	0.9923 ± 0.0101
	Kendall τ (mean ± std)	0.9648 ± 0.0286

The indicator-oriented evaluation shows that the proposed forecasting outputs preserve both short-term temporal dynamics and decision-relevant spatial prioritization. The relative-change errors remain moderate, with Δ*y* MAE and RMSE of 2.6894 and 5.0440 across all provinces, and only a slight increase to 2.8540 and 5.2515 within high-burden provinces, suggesting that the model captures month-to-month variation without disproportionate degradation in the most critical regions. Meanwhile, hotspot ranking consistency is highly stable over time, as reflected by Spearman ρ = 0.9923 ± 0.0101 and Kendall τ = 0.9648 ± 0.0286, indicating that the predicted provincial ordering closely matches the ground-truth ordering in most months and that priority hotspots can be reliably identified for surveillance-oriented resource allocation.

## 4 Discussion

This study targets the spatio-temporal forecasting of provincial-level monthly HIV/AIDS case counts and proposes a graph-aware spatio-temporal attention mechanism together with a seasonal structure-aware temporal module for public health surveillance. Across multi-metric evaluations and visualization-based diagnostics, the proposed approach exhibits more stable overall behavior. From a discussion perspective, the significance of these improvements lies not only in reducing average errors, but also in making prediction biases more uniformly controllable across space and time, thereby lowering the risk of systematic overestimation or underestimation in specific high-burden regions or critical months. For public health governance, the value of short-term forecasting extends beyond numerical accuracy, insofar as it supports anticipatory planning, prioritization and the efficient allocation of limited resources across jurisdictions. From an implementation perspective, the framework is compatible with routine monthly reporting workflows because it only requires regularly updated case-count tables and a fixed spatial boundary file, making it feasible to run as a lightweight analytical component within existing surveillance pipelines. A practical integration pathway is to deploy the trained model as a scheduled service that ingests newly reported monthly data, performs the same preprocessing steps, and outputs province-level forecasts and ranked risk summaries to a dashboard used by surveillance staff.

For public health practice, more reliable short-term forecasts can support finer-grained proactive resource allocation, such as scheduling of testing supplies and clinical services, prioritizing risk communication and interventions in key areas, and issuing earlier alerts for seasonal fluctuation periods. Moreover, introducing graph-structured cross-regional dependencies is more consistent with plausible assumptions about real-world mobility and transmission pathways, which can facilitate earlier identification of synchronous increases or asynchronous rebounds across regions in macro-level surveillance. Such outputs can be combined with threshold-based or rule-based alerts by triggering warnings when predicted counts exceed historical baselines or when multiple neighboring provinces exhibit concurrent increases, thereby complementing existing indicator monitoring rather than replacing it. Key operational challenges include ensuring consistent data formatting across reporting sources, maintaining reproducible pre-processing, handling late revisions of historical records, and selecting alert thresholds that balance sensitivity and false alarms under different resource constraints. Experience from the post-pandemic period has shown how health systems may need to reorganize care pathways in response to rapidly changing demand and constraints (19) and how longer-term morbidity and rehabilitation needs can translate into sustained service pressure if not anticipated early (20, 21).

In parallel, work using population-level immunological benchmarks has illustrated how external reference metrics can support policy-relevant inference and highlight hidden vulnerabilities (22). Together, these perspectives reinforce the manuscript's core rationale that surveillance analytics should be designed to inform timely, proportionate, and operationally actionable decisions, rather than being treated as purely technical forecasting exercises. To support decision use, the system can further provide uncertainty-aware outputs such as prediction intervals or calibrated risk scores, and incorporate human-in-the-loop review to validate alerts before action, which is important when forecasts influence resource allocation and public communication. Practical deployment should also consider data governance and access control, including secure handling of surveillance records, audit trails for model outputs, and clear documentation to support reproducibility and accountability in routine public health operations.

Nevertheless, provincial-level aggregated data entail unavoidable limitations. First, reported case counts are affected by testing coverage, reporting delays, changes in diagnostic standards, and policy adjustments, meaning that observed sequences may contain structural shifts not primarily driven by epidemiological dynamics. In addition, routine surveillance data may include under-reporting, delayed backfilling, and occasional administrative corrections, which can introduce non-stationary artifacts and inflate apparent variability in certain months or provinces. Although the proposed model improves robustness, its performance still depends on the stability of reporting practices and on the degree to which notification counts reflect true incidence rather than surveillance intensity. Second, aggregation at the provincial scale can mask within-province heterogeneity, preventing the model from directly characterizing finer-grained hotspots and high-risk populations. Third, graph structures approximated by administrative adjacency or other static relations may not fully reflect dynamic changes in inter-provincial population movement, healthcare accessibility, and socioeconomic connectivity. Moreover, the adjacency-based graph is a proxy for interaction and may misrepresent long-distance connectivity driven by migration corridors, transportation hubs, or intercity referral patterns. Finally, the current study focuses on one disease and one national context, and generalization to other infectious diseases or countries may require careful adaptation because diseases differ in transmission routes, seasonality strength, reporting processes, and spatial coupling mechanisms, while administrative units and healthcare systems also vary across settings. Therefore, future work can, without altering the task definition, incorporate exogenous covariates and dynamic graph construction strategies that more closely align with public health mechanisms, such as mobility intensity, key public events and intervention timestamps, testing volume, and reporting-delay corrections. For broader applicability, future extensions can evaluate cross-disease transfer and cross-country portability by adjusting the graph definition to reflect local mobility or referral networks and by re-estimating seasonal components under disease-specific periodicities. In operational deployment, updating strategies are also needed to accommodate concept drift caused by policy changes or intervention rollouts, for example through periodic retraining, rolling-window validation, and monitoring of forecast residuals for systematic bias. In addition, strengthening error calibration and uncertainty expression for critical months and high-burden regions would make the forecasts not only more accurate, but also more suitable for directly supporting surveillance decision-making and risk communication.

## 5 Conclusion

This paper addresses the spatio-temporal forecasting demand for provincial-level monthly HIV/AIDS case counts by developing a graph-centric predictive framework. On the spatial dimension, a graph-aware spatio-temporal attention mechanism is introduced to explicitly model inter-provincial associations and cross-regional propagation pathways, while on the temporal dimension, a structured sequence representation is designed to strengthen the characterization of long-term evolution and abrupt fluctuations, thereby enabling more robust inference of future case-count distributions. Under a unified data processing pipeline and evaluation protocol, the proposed method demonstrates consistently better performance on both error-based and structure-similarity metrics, and exhibits a more uniform bias distribution in visualization-based diagnostics such as the month–region error heatmaps, indicating that it can maintain more reliable forecasting quality in high-burden regions and critical time windows. Overall, this study provides an implementable spatio-temporal forecasting tool for public health surveillance, which can support risk early warning, resource allocation, and prioritization of interventions in key regions, and establishes a methodological foundation for future integration of richer public health drivers and enhanced interpretability-oriented evaluation.

## Data Availability

The original contributions presented in the study are included in the article/Supplementary material, further inquiries can be directed to the corresponding author.
